# Twin-Twin Transfusion Syndrome After Radiofrequency Ablation in Dizygotic Monochorionic Triamniotic Triplet Pregnancy With Sex Discordance: A Case Report

**DOI:** 10.3389/fmed.2022.924356

**Published:** 2022-06-30

**Authors:** Mingming Tang, Xingbo Tian, Jianglai Luo, Xiaohang Zhang, Suzhen Ran, Jiaojiao Xiong, Yanlin Chen, Gongli Chen

**Affiliations:** ^1^Department of Perinatal Surgery, Chongqing Health Center for Women and Children, Chongqing, China; ^2^Department of Ultrasound, Chongqing Health Center for Women and Children, Chongqing, China; ^3^Department of Prenatal Diagnosis, Chongqing Health Center for Women and Children, Chongqing, China; ^4^Department of Pathology, Chongqing Health Center for Women and Children, Chongqing, China

**Keywords:** dizygotic triplet pregnancy, monochorionic triamniotic triplet pregnancy, radiofrequency ablation (RFA), twin-twin transfusion syndrome (TTTS), sex discordance

## Abstract

**Background:**

We report a case of dizygotic monochorionic triamniotic triplet pregnancy. Twin-twin transfusion syndrome was subsequently diagnosed combined with sex discordance in the two surviving fetuses after one fetus was reduced, which is extremely rare and has not been previously reported.

**Case Presentation:**

After reducing one fetus by radiofrequency ablation of a monochorionic triamniotic triplet pregnancy, twin-twin transfusion syndrome was subsequently diagnosed combined with sex discordance in the two surviving fetuses. Amniotic fluid for chromosome analysis showed normal karyotype 46, XY/46, XX of the donor and recipient fetus, and short tandem repeat (STR) analysis revealed dizygotic twins.

**Conclusions:**

Through this is an unusual case, we aim to emphasize the importance of accurate diagnosis of chorionicity and zygosity in sex discordant triplet pregnancy, which is the key to appropriate clinical management.

## Introduction

Since a series of cases of monochorionic dizygotic (MCDZ) twin pregnancies have been reported (1–6), more attention has been given to sex-inconsistent twin pregnancies in practice, which may be associated with the increased use of assisted reproductive technology (ART) (1). However, MCDZ triplet pregnancy with sex discordance has rarely been reported.

Triplet pregnancy (7) increases not only the incidence of maternal complications but also the incidence of abortion, preterm birth, perinatal death, cerebral palsy and other complications, seriously increasing the burden on families and society.

Monochorionic multiple gestations are related to an increased risk of fetal and neonatal complications. Twin-twin transfusion syndrome (TTTS) occurs in 8% to 15% of monochorionic (MC) twin pregnancies (2), resulting in an imbalance of the blood flow due to anastomosis of the placental communication vessels. Fetoscopic laser surgery (FLS) is considered to be the preferred treatment for selected cases (1), which blocks the process of the disease by clotting the placental communication vessels.

Here, we report an unusual case of dizygotic monochorionic triamniotic triplet pregnancy. After reducing one fetus by radiofrequency ablation (RFA), twin-twin transfusion syndrome was subsequently diagnosed combined with sex discordance in the two surviving fetuses.

## Case Description

**Figure 1:**
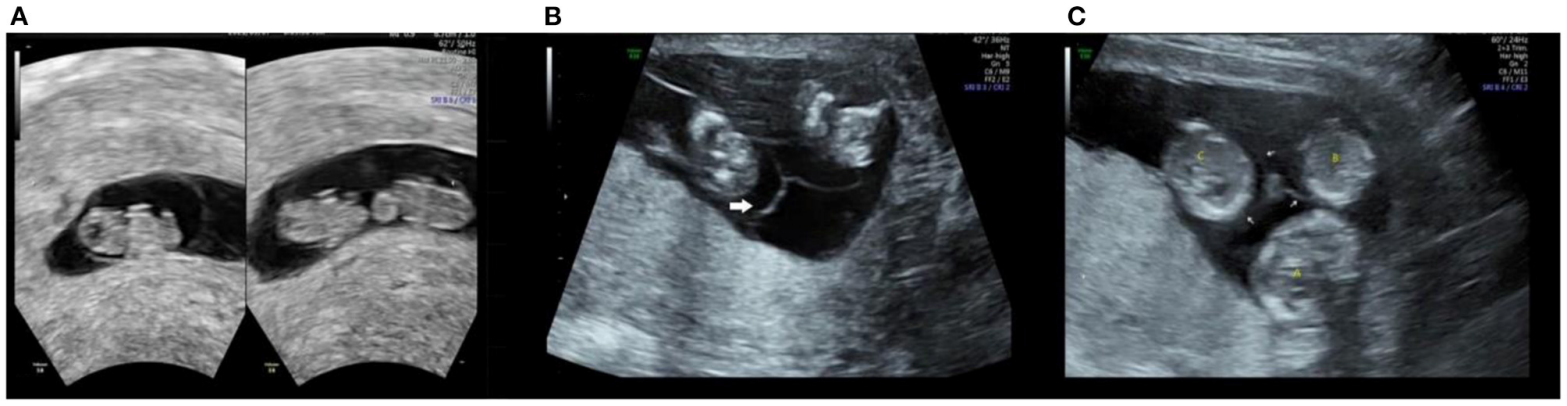
Ultrasound image during the first trimester. At the 59th days of pregnancy, ultrasonography shows one gestational sac with three yolk sacs **(A)**. At 13 0/7 weeks of gestation, fetuses **(A–C)** are separated by intertwin membranes (arrow).

The patient was 36-year-old gravida 2 para 1, last menstrual period (LMP) was July 11, 2021, who conceived through ART on July 28, 2021, and two embryos were transferred. Early ultrasonography indicated the diagnosis of monochorionic triamniotic triplet pregnancy at 13 0/7 weeks of gestation (Figure 1), the crown-rump length (CRL) was 73.6/67.4/72.3 mm and the nuchal translucency (NT) was 1.5/1.4/1.3 mm respectively. Considering a history of cesarean section and the high risks of continuing triplet pregnancy, the patient and her family decided to reduce one fetus after being fully informed about the risks of keeping one or two fetuses for subsequent pregnancy. After obtaining signed informed consent, we performed radiofrequency ablation at 17 5/7 weeks of gestation to reduce one fetus with umbilical cord velamentous insertion (leaving one normal fetus and the other with a single umbilical artery) and extracted the amniotic fluid for analysis from the single umbilical artery fetus during the operation due to the advanced age of the patient. The patient was discharged 3 days after surgery without complications and followed by ultrasound assessment.

**Figure 2:**
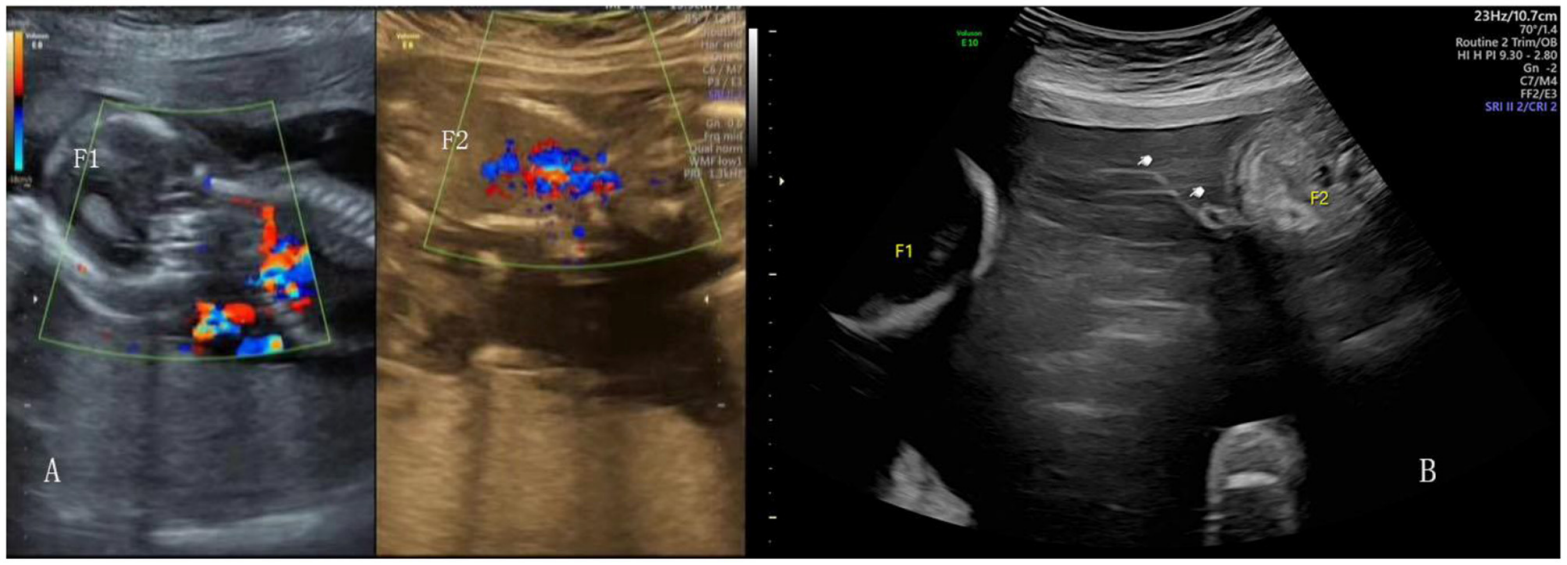
Ultrasound image of the surviving twin after RFA **(A)**. Ultrasound image of TTTS, the donor amnion (white hand) floating in the amniotic cavity **(B)**.

Twenty-four days after RFA surgery, ultrasonography confirmed the diagnose of twin-twin transfusion syndrome (Figure 2) and discordant fetal sex in the two surviving fetuses. We rechecked the chorionicity and the reduced fetus was male. The male donor twin (the one with a single umbilical artery) had an amniotic fluid maximum vertical pocket of 1.7 cm, and the female recipient twin 12.1 cm with ductus venous reversed flow, severe TV regurgitation and pulmonary regurgitation, absent bladder was found in both fetuses. Selective fetal growth restriction (sFGR) was also achieved, as the fetal weight difference was 44 percent and the smaller twin (donor twin) weight was <10th percentile. After discussing all management options, the patient and her family chose FLS and signed informed consent. Operative fetoscopy and Solomon surgery were performed at 21 1/7 weeks of gestation, and the amniotic fluid of the recipient twin was sent for analysis. During the surgery, we found that the male donor fetus and the female recipient fetus had normal-appearing external male and female genitalia respectively. Two large arteriovenous and several small communication vessels were identified and ablated, and a continuous laser photocoagulated the placental surface, connecting the previous ablated points.

**Figure 3:**
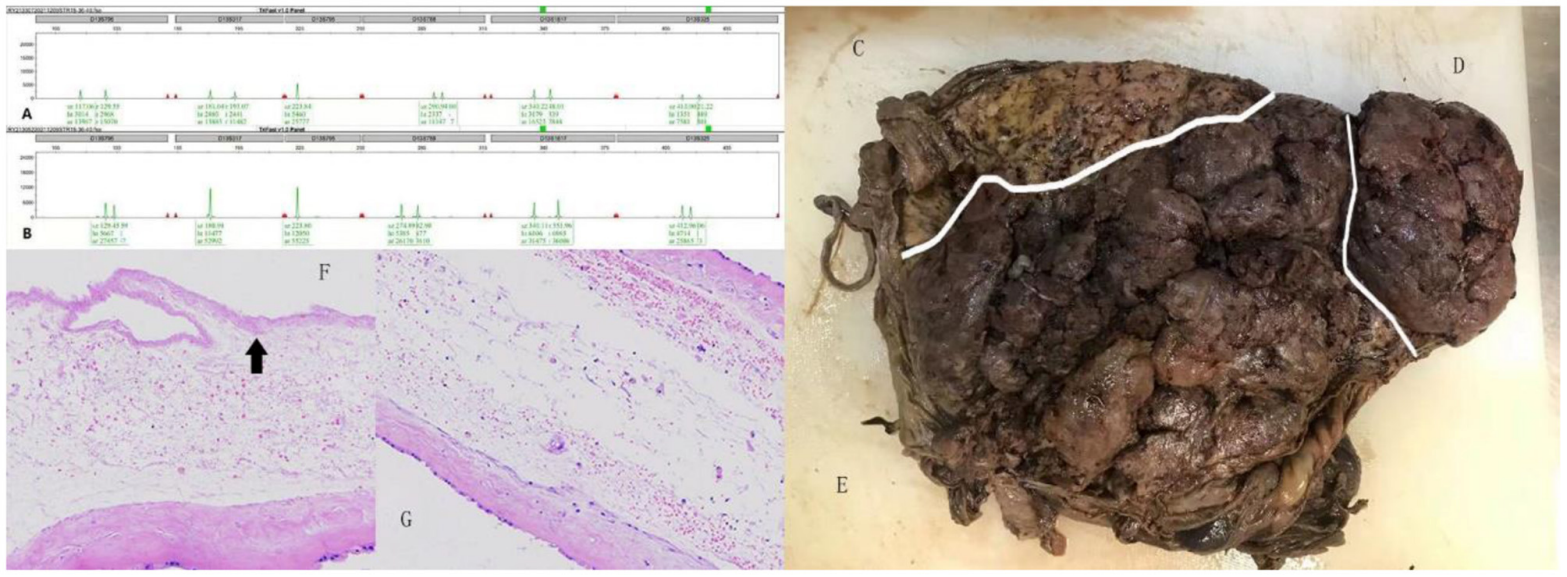
**(A,B)** Indicate the genotypes of the female recipient twin and the male donor twin at different loci, respectively. Because both alleles were different, the twins were accepted as DZ. Gross placental specimen appearing of the reduced male fetus **(C)**, the male donor twin **(D)** and the female recipient twin **(E)**, separated by white line respectively. Histological examination of intertwin membrane between the reduced male fetus and the female recipient twin **(F)**, the female recipient twin and the male donor twin **(G)**, reveals MC component. The black arrow showing cytolysis of amniotic epithelial cells of the reduced male fetus.

The amniotic fluid for chromosome analysis revealed a normal male karyotype (46, XY) of the donor twin and normal female karyotype (46, XX) of the recipient twin, and short tandem repeat (STR) analysis revealed dizygotic twins (Figures 3A,B). Unfortunately, the donor male fetus died *in utero* on the first day after surgery. The patient was delivered at 30 4/7 weeks by cesarean section. The girl's (recipient twin) birth weight was 2000 g with normal external female genitalia appearing and the other two male stillbirths showed external male genitalia. Pathological examination confirmed the diagnose of monochorionic triamniotic pregnancy (Figures 3C–G).

## Discussion

Considering the high risks, we began to communicate with the patient and her families about reducing fetus at the time the diagnosis of monochorionic triamniotic triplet pregnancy was made (13 0/7 weeks of gestation). However, the patient and her families were hesitant and not admitted to hospital for reducing fetus until the second trimester (17 5/7 weeks of gestation), which may have caused the interaction between the three fetuses to some extent. Considering the poor prognosis of TTTS combined with sFGR, we suggested that reduce the smaller twin (donor twin) might be a better choice, but the patient and her family firmly refused to reduce the fetus and requested FLS to keep twins.

It is generally viewed that MC twin pregnancy is monozygotic. Since 1970, Nylander and Osunkoya reported an unusual monochorionic placentation with sex discordant twins (6), an increasing number of clinical cases of MCDZ pregnancies have been reported (1–6).

In our case, according to the monozygotic twin development theory, we speculate that the trophoblasts from 2 different blastocysts fused within the first 72 h after fertilization, and the male blastocyst division occurred between the fourth and eighth day, finally developing into a dizygotic monochorionic triamniotic triplet pregnancy combined with sex discordance.

One remarkable observation that we discovered in our case is the significance of the identification of chorionicity and zygosity in sex discordant triplet pregnancy in clinical practice management. Monochorionic multiple pregnancy shows an increased risk of fetal and neonatal complications, such as TTTS and twin anemia–polycythemia sequence (TAPS). Although TTTS occurs in 8% to 15% of monozygotic monochorionic twin gestations (2), it should be taken into consideration when dealing with monochorionic triamniotic triplet pregnancy in clinical practice if two fetuses are kept.

Twins with opposite sex are almost dizygotic. However, it can also occur in monozygotic twins (8). Most reported cases (9, 10) described female twin with Turner syndrome (45, X), resulting from loss of the Y chromosome in 46, XY twin. Zech et al. (11) found a rare case of a 47, XXY zygote that underwent postzygotic loss of the X chromosome in some cells and Y chromosome in other cells, resulting in the phenotype of the resultant twins was one male and one female. Monogenic disorders can affect sex discordance (12) and malformed external genitalia can also occur unrelated to chromosomal or genetic disorders (13, 14).

In conclusion, we report a prenatal case of dizygotic monochorionic triamniotic triplet pregnancy. After reducing one fetus by radiofrequency ablation, twin-twin transfusion syndrome was subsequently diagnosed combined with sex discordance in the two surviving fetuses. Our report aims to emphasize the importance of an accurate diagnosis of chorionicity and zygosity in sex discordant triplet pregnancy, which is the key to appropriate clinical management.

## Data Availability Statement

The original contributions presented in the study are included in the article/Supplementary Material, further inquiries can be directed to the corresponding author.

## Ethics Statement

Written informed consent was obtained from the individual(s) for the publication of any potentially identifiable images or data included in this article.

## Author Contributions

MT analyzed patient data and drafted the paper. XT and JL collected patient data. XZ, SR, JX, and YC provided related patient clinical information. GC provided ideas and revised this paper. All authors read and approved the final manuscript.

## Conflict of Interest

The authors declare that the research was conducted in the absence of any commercial or financial relationships that could be construed as a potential conflict of interest.

## Publisher's Note

All claims expressed in this article are solely those of the authors and do not necessarily represent those of their affiliated organizations, or those of the publisher, the editors and the reviewers. Any product that may be evaluated in this article, or claim that may be made by its manufacturer, is not guaranteed or endorsed by the publisher.
